# Collectanea

**Published:** 1831-11

**Authors:** 


### COLLECTANEA.

**Floriferis ut apes in saltibus omnia libant,**

**Omnia nos, itidem, depascimur aurea dicta.**

### PHYSIOLOGY.

*Regeneration of the Nervous Tissue.* M. PREVOST, having selected five young cats, divided in each the left pneumo-gastric nerve, and removed a portion of about six millimetres.* No symptoms ensued, and the wound speedily healed. A month afterwards, Mr. P. divided the right pneumo-gastric in one of these cats: frequent yawning followed, and hoarse cries expressive of great pain; the respiration was impeded; and in fifteen hours death took place. Upon examination, the two extremities of the left pneumo-gastric nerve were found swollen, and united by means of a whitish tissue, which looked like thickened neurilema.

A month after, the same experiment was tried on a second of the cats, and similar phenomena were observed, excepting that death did not occur for thirty-six hours.

In two months more, the right pneumo-gastric of a third cat was divided: no inconvenience appeared to be caused. The same experiment was tried on the fourth cat, with a similar result. The fifth cat had died by accident.

In fifteen days, the right pneumo-gastric was again divided in the two last animals, a little above the part where it had at first been divided: still the breathing was not affected. In thirty-six hours after, the nerve on the left side was divided in each, just above where the first section had been made, and in thirty hours both the cats died. The dissections of the divided nerves proved that the upper and lower ends of the left pneumo-gastric were firmly connected, and that the point of reunion was marked by a whitish and hard enlargement, which appeared to be covered by a layer of thickened neurilema. Upon removing this external layer, the central part was pressed between two pieces of glass, and examined with a microscope; and it was easy to discern filaments of the trunk of the nerve passing through the interposed substance, from the upper to the lower end, by which the integrity of the nerve was re-established. These filaments were not in a regular juxtaposition, as they are in a continued nerve: they were separated, and appeared as though they had with difficulty penetrated the intermediate substance.

M. Prevost concludes, 1st, that, when a nerve has been divided, the reunion of the two ends, which soon takes place by means of a whitish cellular tissue, is not sufficient to re-establish the function of the nerve. 2d. That it is necessary that this interposed substance should be traversed by nervous filaments extending from the upper to the lower divided portion. 3d. That this restoration appears to require a considerable period of time before it is perfect.—*Annales des Sc. Naturelles.*

### PATHOLOGY.

*Narcotism from Absorption;* by M. GERHARD. In the month of May, 1830, the child of the Countess of R. was suddenly seized with symptoms of a very serious nature. The ordinary physician being from home, I was called. The child, two months old, had been up to this period in good health: on my arrival, I found her in the following state: Colour of the face not much changed; eyes dull and fixed, their pupils more dilated than natural, and shewing little sensibility to light; respiration slow, and the motions of the chest scarcely discernible; the pulse obscure and very slow; the belly slightly

* A millimetre is .03937 of an English inch.—EDITOR.

tympanitic. The child had been insensible for some hours, and was incapable of swallowing any food put into her mouth; she uttered not a cry, except when smartly pinched, and there were occasional slight convulsive motions, especially of the muscles of the face and mouth.

While inquiring into the cause of these symptoms, which did not appear to be the result of any pre-existing organic affection, I perceived that the skin of the root of the neck was excoriated, all along a wrinkle extending round its front to either side, and that this part, as well as the linen which covered it, was stained of a yellow colour. I then learnt that the excoriation having for several days caused the child very severe pain, the physician had ordered it to be dressed with a mixture of cerate and laudanum; and I discovered from the prescription, dated the preceding day, and from what remained of the mixture, that the skin of the neck had been for twenty-four hours in contact with about fifteen drops of laudanum. Every thing was then explained: I had to treat an excess of narcotic action, by which the brain was principally affected. The absorption of the laudanum had been favored by the excoriated state of the neck. I removed with the greatest care the cerate and laudanum which covered the neck, and ordered four leeches to be applied to the lower part of the belly. Two hours after a moderate flow of blood, the face became a little pale, but the vital functions began to rally: the child could swallow a few spoonfuls of gooseberry water, and this was continued every quarter of an hour. Five hours after my arrival she began to suck, but feebly; through the night, however, she took the breast frequently. During the next day, the alvine evacuations, which had been suspended, resumed their natural state, and the symptoms of narcotism were rather diminished, but they did not wholly disappear till the third day after their attack.—*Journal de Chimie Med.*, and *Glasgow Med. Examiner.*

---

*Death from Inanition. Some Account of Granet, the French Prisoner, who lately starved himself to Death.* The following brief report was transmitted to the French Academy of Medicine, by the Minister of the Interior. It was drawn up, we understand, by M. Dufour, a physician of Toulouse, who attended the prisoner during the whole period of his sufferings and death.

Guillaume Granet was twenty-four years of age. It was on the 20th of April last that he formed the fatal resolution of starving himself, with intent to avoid the punishment due to his crimes. On the 21st he was found lying on the ground, dressed, head uncovered, his irons bolted on both hands and feet; he would only answer by signs, and would take neither solid nor liquid food. It was attempted to make him swallow by force; but this only threw him into an outrageous passion. When visited in the evening, however, he was more calm, and thought fit to use his tongue.

22d and 23d. Much the same way; urine fœtid; burning heat in the throat.

24th. Face flushed; breath exceeding foul; haggard looks; pulse at the wrist scarcely perceptible.—25th. He passed some urine.

26th and 27th. Nothing remarkable.

28th. He came down to the court-yard, and drank up some water from the well: his handcuffs taken off.

29th. Shiverings.—30th. Drank a glass of water.

May 1st. Spoke, but not to be understood.

2d. Rolled himself in the kennel of the court-yard.

3d. Drinks some water; will take no broth or soup.

4th. Passes some urine. On the 5th, he ran out of his dungeon, with nothing on him but his shirt: upon coming back, he lay down and ate a morsel of bread. He was taken to the infirmary, and about midnight ate some soup, with a little bread and wine.—6th. Took nothing.

7th. Drank of his own urine, and about a quarter of a spoonful of water. In the afternoon he complained that there was poison in the food that was offered him.

On the 9th, 12th, 15th, and 17th, he drank some water.

18th. Drank but half a spoonful; attacked with violent fits of coughing, and oppression of the chest.

21st. Ran out, and wanted to drink of the kennel, but was prevented; same night he drank water.

22d. Wanted to bite and scratch; complained of sharp pains in his bowels; passed water.—23d. Took some broth.

On the 24th, he was found lying on his belly; his pulse feeble and slow; his fists shut: drank some water from the kennel, which had been cleansed.

25th. Suffered much, and abused every body; pulse fifty-three; drank of the kennel.

26th. Tore his shirt to pieces; same night passed some urine.

On the 27th and 28th, he drank eight glasses of water; his fæces were found carbonized; said he had still a fortnight to live.

In this way, at one time easy and again in agony, now refusing to drink and now taking a little water, he continued to live on till the 17th of June (the fifty-eighth day,) when, about five in the morning, he died, after struggling for four hours in convulsions.

During some of his last days he complained of cold; his legs too were shrunk up, and marked with blackish spots: dry gangrene, according to M. Dufour.

These are all the facts of this wretched man's suffering which have been preserved. The particulars of his dissection have not yet reached the Academie, but are promised to be sent ere long by the minister.—*Med. Gazette.*

---

*Remarkable Instance of Hemorrhagic Tendency in a Family. (Medizinisch-Chirurgische Zeitung.)*

Various instances have been published in this Journal, as well as in others, more particularly on the continent, of the hereditary transmission of the hemorrhœal diathesis through several individuals in a family. To those previously published, a very complete example has lately been added by Dr. Riecken, of the principality of Birkenfeld in Oldenburg, in a publication entitled "New Researches relative to the Hereditary Tendency to Fatal Hemorrhages." In this work, of which a full analysis is given in the journal quoted above, some general observations are made upon the etiology of the hemorrhagic tendency, and on the proper mode of counteracting it. But the most interesting part of its contents is a minute history of the family whose melancholy peculiarity of constitution led the author to turn his thoughts to the subject.

The father of the family, Ernest P., was a husbandman and joiner, who had always enjoyed good health, and at the time of the publication of Dr. Riecken's work in 1829, was in his eighty-sixth year. His second wife, by whom he had the children to be mentioned presently, was of delicate health. In her thirtieth year she was attacked with rheumatic gout, and, after this ceased, with curvature of the spine, asthmatic complaints, and frequent pain under the breast bone. By and by signs of water in the chest came on, general dropsy followed, and she died of that disease in her sixty-sixth year. Neither the wife nor the husband was ever subject to hemorrhage, or to petechial spots, This couple had twelve children, five sons and seven daughters, of whom four died of smallpox, one of eclampsia, and three boys and one girl of hemorrhagy.

William Louis, one of the three boys, enjoyed good health till his fourth year, when he was attacked with bleeding from the left nostril, which continued, with occasional intermissions, for eight days, and was only arrested by stopping up both nostrils firmly with the Boletus Igniarius *(zündschwamm.)* Two days afterwards, he was seized with anxiety and a sense of constriction in the præcordia, attended by swelling and tenderness there, then with cold sweating and deadly paleness, and at length with vomiting of black fluid blood, which repeatedly returned, and proved fatal in the course of a week. In another of the boys, John Christian William, blue spots, unattended with pain, frequently broke out on the skin between his first and eleventh years, but were not accompanied by hemorrhage. In his tenth year he was attacked with violent rending pain in the extremities, more especially in the limbs, which abated in a month and a half, after a hard tumor formed ou the left knee. This tumor had been present for a year and a half, when he was suddenly seized with violent toothach in the foremost grinder of the left side of the lower jaw, and the pain was so excruciating that he consented to the extraction of the tooth: the tooth was quite healthy. A gush of fluid blood immediately took place from the cavity, and nothing could check it. The poor boy gradually became blanched like wax, and expired on the eighth day. Philip Henry, the third boy, presented the appearance of blue spots on the skin, particularly of the buttocks, even during the first year of his life, yet without any signs of weakness. When a year and a quarter old, he died while vomiting fluid blood, which had begun the day before, without any previous appearance of ill health. The daughter died four days after birth, in consequence of hemorrhage after the division of the frœnum of the tongue.

The same striking constitutional infirmity likewise appeared among the grandchildren of Ernest P., born of his youngest daughter, Lousia Catharine. This woman, who is still alive, and in the thirty-second year of her age, is short in stature and light haired, has grey eyes, and a delicate, fair complexion. She never had purple spots, or exhibited any tendency to hemorrhage; nay wounds, and even apertures in veins made by the operation of bloodletting, healed in the usual manner. The menses commenced in her thirteenth year, and were usually rather abundant, and of eight days' continuance. She suffered much from toothach and neuralgic affections of the extremities, was indeed seldom altogether free of wandering pains, and in her pregnancies was so much affected by a tendency to plethora that it was frequently necessary to withdraw blood. The blood coagulated more slowly than usual, was very dark, contained much serum, and presented a thin buffy coat. The hemorrhage immediately after delivery, as well as the lochial discharge, was always profuse. Her husband is a stout, healthy man. They have had six children, four boys and two girls, of whom only the eldest girl and youngest boy are now alive. The remaining girl died of convulsions when nine months old, and the three boys of hemorrhage. The surviving girl is very healthy, and never had either blue spots or bleedings.

The four boys were all born easily, and the navel healed up without hemorrhage. They all had a disproportionately large head, with unusually loose sutures, and fontanelles of uncommon size and slowly filled up. Their bodies were delicately and regularly formed, the nails were of the natural appearance, the skin fair and delicate, with the veins shining distinctly through, and the countenance pale, sickly and bloated. They had all blue eyes, and one of them fair, but the three others black hair. Dentition went on, in all, in the usual manner. They were very lively, of mild dispositions, and the eldest shewed much cleverness. The operation of vaccination, which was performed by incisions, was not followed by any untoward effects. All had from birth a very fetid discharge of a white, flaky, puriform mucus from each ear.

The eldest of these boys manifested a distinct tendency to hemorrhage in the seventh month: dark, irregular spots appeared on various parts of the skin, varying in size from that of an *achtgroschen* piece to that of half a man's hand, and without any external injury; and these were at first pale red, but as they increased in size rapidly became bluish black, and then reddish blue, bluish green, and dirty yellow as they were disappearing. At times the body was entirely covered with them. They were attended with hardness and swelling, but not with pain. The first attack of hemorrhage occurred in his first year, in consequence of his having bitten the tip of his tongue; and it was not arrested till various artificial means had been tried in vain, and nature accomplished it after the child was reduced to the lowest possible state of exhaustion. When he was eighteen months old, a second attack took place in the form of epistaxis, which was not checked till he was almost at the point of death; when it ceased under the use of the acid elixir of Haller and laudanum, and during the deep sleep which supervened. On his awaking, general convulsions attacked him; then deep sleep returned, and after this he awoke refreshed, and soon recovered. From this period till he was four years and a half old, he had regularly every three months an attack of epistaxis from the left nostril, which continued between four and ten days with occasional intermissions, and was preceded by lancinating headach, sounding in the ears, excitement of the pulse, flushing of the face, and lividity of the lobes of the ears. The blood was dark-red, thin, without tendency to coagulation, and towards the close of the paroxysm pale dirty-red in colour. The hemorrhage never ceased till the child, after repeated fainting fits, was brought almost to the point of death. In his fourth year he complained much of shifting pains, particularly in the left thigh, which were particularly troublesome before the customary bleedings or on any change of weather, but became much less so on a swelling of the knee-joint making its appearance. This swelling confined him for some time to bed, but it was diminished by proper remedies; upon which a fresh attack of hemorrhage occurred, and after this the pains ceased. The child, however, was pale and exhausted, and in two days he died with all the symptoms of inflammation of the bowels. Sulphate of soda had no effect in checking the hemorrhagies in this case.

In the second boy, the blue spots began to appear fourteen weeks after birth. When ten months old, a furuncle formed in the right armpit, which was carefully opened. At first pus alone issued; but afterwards violent hemorrhage ensued, which continued for three days, notwithstanding the constant use of tents and compresses dipped in alum. After the lapse of nearly three quarters of a year, an almost fatal hemorrhage followed a trifling injury of the frœnum of the lower lip; and this was not checked till on the third day the actual cautery was resorted to. After this, with the exception of the blue spots, the child was healthy, and became plump and strong. But when two years and a half old, plethoric symptoms began to shew themselves, as in his older brother's case, and he was attacked with pleuro-peripneumony. Dr. Riecken avoided all evacuations of blood, and brought him through this illness by other antiphlogistic remedies. Suddenly, however, after a return of fever, copious bleeding took place from both nostrils, for which all the usual means, including Glauber's salt, were in vain put in requisition; and it was not till the child had repeatedly fainted, and was as pale as a corpse, and till the blood had in the end flowed for six hours as pale as bloody serum, that the hemorrhage ceased spontaneously. Half a year afterwards he was attacked by flying pains in the extremities, followed by swelling in the left ankle, and, when this disappeared, by swelling in the left knee. Recovery, however, was gradually so far accomplished, that the child could walk again, when, in an accidental fall, a small wound no bigger than a pin's head, was inflicted on the point of the tongue. Profuse hemorrhage commenced, and for five days it continued, although every conceivable remedy was tried, including three applications of the cauterizing iron, till at length the breathing and pulse ceased, the skin became icy cold, the eyes lost their lustre, and death was believed to be at hand.

After a time, signs of animation appeared; the hemorrhage was found to have ceased, and the child became convalescent. In fourteen days he began to complain of occasional stitches under the ribs of the left side, accompanied with dry cough; and during a fit of coughing, blood began to gush from both nostrils. The blood was fluid, thin, brownish in colour, and fetid. It continued to flow in spite of every remedy which could be thought of, and the little patient soon died slightly convulsed.

The third boy, in consequence of the frœnum of the tongue being unskilfully divided, was attacked when three months old with profuse hemorrhage, which lasted for three days, and only yielded after repeated applications of the actual cautery. The blue spots did not begin to shew themselves till the sixth month. The plethoric symptoms observed in the two former cases appeared also in the present instance about the thirteenth month, and especially some weeks before his death, which took place after an attack of hemorrhage of two days' continuance, occasioned by an injury of the tip of the tongue with an incisor tooth. On this last occasion repeated cauterization was of no use.

In the youngest boy, who is still alive, a chronic itchy eruption of the face was added to the discharge from the ear, which he had in common with his brothers. Blue spots began to appear on the skin four months after birth. But subsequently to the administration of ass-colt liver-oil (!) to the mother, both the eruption and the livid spots disappeared. Whenever the mother intermitted the oil, the spots reappeared; and whenever she resumed it, the discharge from the child's ear dried up, the spots ceased to form: nay, on one occasion he sustained a wound of the ringfinger with a sharp knife, yet the hemorrhage was not greater than in other children. Subsequently a furuncle on the shoulder was opened, and a second time a wound accidentally inflicted without any particular hemorrhage. The further history of this case is not given.

Besides the mother of this family, Ernest P. had two other daughters, who, together with their families, never suffered from hemorrhage or ecchymosis; but they were very liable to gout, rheumatism, chronic abscesses, and eruptions. Among his ancestors and collaterals there was no one ever liable to hemorrhage; nor can the least relationship be traced between his family and any of the previously ascertained instances of families possessing this constitutional infirmity.

The author in his general remarks seems disposed to ascribe the constitutional hemorrhagic diathesis to a gouty taint; but this is surely an erroneous idea, otherwise it ought to be much more frequently met with. In the present, as in every previously recorded instance, it is very remarkable that the infirmity was confined to the male branches of the family, and that, nevertheless, it was transmitted through the female branch to the males of the next generation.—*Edinburgh Med. and Surg. Journal.*

---

*A Case of probable Dislocation of the Heart from external Violence.* By William Stokes, m.d., one of the Physicians to the Meath Hospital, and County of Dublin Infirmary, Corresponding Member of the Medico-Chirurgical Society of Berlin, Honorary Member of the Hunterian Medical Society of Edinburgh.

Mr. B—, aged twenty-one, had enjoyed uninterrupted health until the 7th of May, 1822, when he was severely crushed between one of the arms of a water-wheel of great size, and the embankment on which the axle was supported. Some of his companions had been amusing themselves by entering between the arms of the wheel, and causing it to revolve by their weight; the wheel had been for some time stationary, and he was in the act of following his companions, his legs being already within the body of the wheel, when it revolved. He was thrown on his face upon the embankment, and received a blow from the arm of the wheel, in a line running from the inferior angle of the left scapula to the top of the right shoulder. On its rebound, he fell within the wheel, from which he was immediately extricated. He remained for upwards of three hours in a state of complete insensibility.

As soon as an examination could be made, the following injuries were discovered: Two ribs in the lower portion of the left side, and right clavicle and humerus, and the fifth, sixth, and seventh ribs on the right side, were broken. The right side of the face and chest was emphysematous, and there was complete paralysis of motion in the right arm, with considerable loss of sensation. The patient felt great pain in the right side of the chest, with a sensation as if a foreign body preventing respiration had been introduced into the right lung: the pain was accompanied by violent throbbing and heaving; and it was soon discovered that his heart was pulsating at the right side of the sternum. He had a short dry cough, but experienced no hæmoptysis, and there was no pain or other symptom of pleuritic inflammation at the left side.*

On the day of the accident, free bleeding was twice performed for the relief of the violent pain of the right side, bandages were employed, and in about two days the subcutaneous emphysema disappeared. During the next month he remained in bed, labouring under a short dry cough, always productive of great aggravation of the pain of the side, which on two occasions was so violent, that it was necessary to use venesection. The blood was uniformly inflammatory. At the end of the month he was able to get out of bed, but during the next eighteen months he experienced frequent returns of the pain, calling for the employment of the lancet. The paralysis then began to disappear, and the use of the arm was gradually restored; at first he could only bend the limb, but was unable to preserve it long in the flexed position. He then returned to his studies, but found that he could not read for any length of time, from defective vision, the letters appearing like black lines, though he could still distinguish objects at a distance nearly as well as ever.

From that period to the present time his symptoms have been the following: The heart continued to pulsate on the right side of the sternum, the pulsation being generally strong and aggravated by mental emotion, exercise, or the occurrence of pain in the side. He never had orthopnœa, but always experienced great difficulty of breathing on exercise, or when he attempted to lie on the left side. The cough has remained ever since, being always worse in winter. Any unusual exercise, such as rapid walking, has invariably brought on a violent fit of coughing, which is preceded and accompanied by a peculiar mawkish taste, a sensation which remains for some time after the cough has subsided. From the first he has found that if the right hand be plunged in cold water, he experiences a strange sensation, which passes up the arm, and is then felt within the right side of the chest; and at the same the arm is spasmodically brought across the front of the thorax. Hot water has produced the same effect, but not so violently. He also found that any cold substance applied to the right side of the chest produced an extreme feeling of suffocation; and hence, on his attempting to bathe in cold water, he has always been forced to return immediately.

During the first three years from the receipt of the injury he found that the use of the smallest quantity of meat invariably brought on vomiting in about a quarter of an hour after it was swallowed; the same effect was produced by other food when taken in large quantity, and ever since that period vomiting always follows the use of food, if taken at a time when the dyspnœa is urgent.

* On this point, so important in the diagnosis of the lesion, the patient is perfectly clear: he states expressly that, since the accident, all the attacks of pain have been on *the right side.*

The act has always been productive of great pain, with a sensation of straining in the right mammary region, and considerable excitation of the heart's action. The pain and straining subside soon after the vomiting is over, but the heart has always continued to palpitate strongly for some time.

His appetite has varied according to the state of his respiration, and hence has been better in summer than in winter. Certain articles of food and drink produce a great oppression in the chest, with wheezing respiration. Those which he particularly specifies are milk, wine, gum, and sugar. Any article of food or drink taken in quantity invariably produces dyspnœa and palpitation. Since the accident, he has every winter experienced several inflammatory attacks, in which he suffers from violent pain in the right side, with great increase of dyspnœa and palpitation; the tongue becomes foul, and he is affected with thirst. These attacks are only to be relieved by bleeding, and he now thinks he has been bled upwards of fifty times. The blood has been always buffed and cupped; and it is a remarkable circumstance, that syncope has has never been produced, even after the loss of so much as thirty ounces of blood at a time. These attacks generally last for a week; but in last November the accession of pain continued for three weeks, which is the longest that he recollects.

In the year 1829, he was advised by an eminent practitioner in this city to try the effects of digitalis. From this remedy he experienced considerable relief, and gradually increased the dose until he could take from six to eight grains of the powder, in a single dose, without the slightest inconvenience. On one occasion he took so much as ten grains, and he assured me, that after using the remedy in the dose of eight grains every night for the space of three months, his pulse was never below eighty; its effect was constantly to relieve dyspnœa and diminish palpitation.

At present, when not labouring under any aggravation of symptoms, his appearance does not differ much from that of a person in the enjoyment of good health. His habit of body is spare but muscular, and the countenance is not expressive of distress. He has a hard sonorous cough, with some mucous expectoration in the morning. In his ordinary state the number of respirations is about thirty in the minute, but this is not the case after exertion or an exacerbation of the bronchitis. Indeed, on the morning when I first saw him his breathing was like that of a person in an advanced stage of laryngitis; but he then had an increase of catarrh, and had walked some distance to my house. When he does not take digitalis, the pulse is generally between 100 and 120, regular in strength, and never intermitting but when he uses the remedy. Its usual frequency is between eighty and ninety.

Having stripped the patient, I made a careful examination of the chest. The right shoulder is depressed, but the right side inferiorly is dilated more than an inch.

The left side of the thorax sounds perfectly clear even to its most inferior portion, and in the situation naturally occupied by the heart. Respiration of the puerile character and mixed with some bronchial rales is to be heard over the entire lung, and is as distinct in the mammary region as in the other portions. The sound of the heart is scarcely audible in the upper part of this side, but neither its sound nor impulse is perceptible below the mamma.

The upper portion of the right lung sounds clear, but from the fifth rib downwards there is complete dulness, and here the integuments are exquisitely sensible. In the upper portion, both anteriorly and posteriorly, the respiratory murmur is of the same character as in the opposite lung, but from the fifth rib downwards it is wanting, except along the spinal column, where it can be heard feebly. There is no bronchial respiration or resonance of the voice.

The pulsations of the heart can be felt and seen in the right mammary region, between the sixth and seventh ribs, and within an inch of the sternum. When not over excited, the sounds of the heart are almost natural, hardly different from those in the healthy state. The impulse precedes the pulse at the wrist by an appreciable interval. There is no sign of valvular disease.

That the heart is displaced in this case does not, as it appears to me, admit of the slightest doubt. Now the circumstances hitherto recognized, productive of this state of parts, are:

1st. Congenital malposition.

2d. The existence of an extensive empyema of the left side.

3d. The growth of tumors in the left cavity of the chest.

4th. Pneumothorax of the left pleura.

5th. Dilatation of the air-cells of the left lung.

In addition to these, we have two other sources of displacement which are much rarer, hernia of some of the abdominal viscera through the diaphragm, and aneurism of the abdominal aorta, a cause first signalized by Dr. Graves and myself.

But the displacement in this case can be easily shewn to depend on no one of these causes. The patient is perfectly clear on the point, that, previous to the accident, he often felt his heart pulsating in the natural situation, and he was the first to point out to his friends that the pulsations occurred at the right side of the sternum after the injury. I questioned him most minutely on this subject, and he assured me that he frequently, after exercise, used to place his hand below the left mamma, to feel his heart beating. Add to this the excellent state of health which he enjoyed previous to the accident, and we cannot suppose that a congenital malposition could have existed. The great rarity of such an occurrence must also be taken into consideration.

I am not aware of any case in the records of modern medicine, in which the symptoms of congenital malposition of the heart in the adult have been observed. The cases detailed by Lancisi, where pulsations occurred at the right side of the chest, were examples of dilatation of the right cavities of the heart. Riolan, however, details two cases of pulsation at the right side of the sternum, in which the patient suffered no inconvenience; but the true nature of these cases is not known, while those mentioned by Senac and Bonetus, are examples of effusion into the left pleura.

That the cause of displacement in the present instance is neither a fluid, solid, nor aeriform collection in the left pleura, a diaphragmatic hernia, nor an aneurism of the abdominal aorta, is at once proved by the examination of the left side by auscultation and percussion. I may remark, that a case more illustrative of the importance of these modes of diagnosis could hardly be found. It might be supposed by some that the pulsations at the right side of the sternum were produced, not by the heart, but by an aneurism of the descending thoracic aorta; but this opinion I think untenable, as we know that, when an aneurism of the descending portion of the aorta gives a double pulsation, it must be of great size, so as to press against the heart. The tumor presenting on the right side, the heart would be pushed strongly against the left ribs, which is not the case here; and I may remark farther, that in such cases we can always feel two separate pulsations, one of the aneurismal tumor, the other of the heart, a circumstance which is wanting in this case.

We must then admit, that this is an example of dislocation of the heart, with rupture of the pericardium and right pleura, a supposition which appears to me to agree perfectly with the history of the case and the present state of the patient. Thus we have an example of displacement of the heart from a cause not hitherto recognized. From the history of the case it appears highly probable that the patient has suffered under repeated attacks of pleuritic inflammation of the right lung. A question now arises, What is the actual state of the lower portion of the right thoracic cavity? Connecting the frequent attacks of pain with the absence of respiration in the inferior portion of the right side, it becomes possible that a circumscribed empyema may exist; but the presence of the heart in the right side of the chest, under such peculiar circumstances, of course renders this supposition problematical. As to the dilatation of the side, this may be produced by the heart itself.

The power which the patient has acquired of bearing such great doses of digitalis is very remarkable. Of course much of this must be attributed to the acquired habit from its long-continued use; but in his case it will be seen that even after taking the remedy for a length of time, the pulse was not brought under eighty beats in the minute. The state of irritation of the heart will partly explain this. How far its displacement is concerned is a matter for future investigation. But the most singular circumstance connected with this extraordinary case, is the fact of the patient, after so dreadful an accident, having lived so long and enjoyed a tolerable state of existence. This renders it probable, that if hypertrophy of the heart exists, it must be slight, an opinion which is borne out by his state at my last examination, when, although he had not used digitalis for many days, the action of the heart was perfectly tranquil. —*Ibid.*

---

### MISCELLANEOUS.

### Mr. Guthrie's *Introductory Lecture, October* 3, 1831. *Extract respecting Professional Education.*

I consider it necessary that every practitioner, without exception, should commence his education by learning pharmacy: six months appear to me to be a sufficient time for this purpose in a well-regulated place, but twelve months may be allowed without disadvantage. When persons have not the means of sending their sons to attend an hospital, a second year may even be occupied in this manner; but all after this is for the advantage of the master, and not of the student; so that when a young man is bound apprentice for six years, four of which are passed behind the counter, and the last two only are devoted to study in the hospital or dissecting room, the middle two years are a period of time nearly thrown away. The regulations of the College of Surgeons unfortunately favor this system, by requiring six years to be passed in some sort of professional study, two years only of which are inquired into, or in which the course of study is regulated. The College, in doing this, have stated the *minimum* of study they think necessary for an individual to undergo; but it has always appeared to me to be a bad regulation: a man is much more competent to practise his profession with advantage to the public who pursues his classical and other general studies until he is seventeen years of age, then learns pharmacy for a year, and during the succeeding four years regularly attends the practice of an hospital, lectures, &c. Five years occupied in this way are infinitely better and more advantageously employed, than six passed in the usual way of apprenticeship; which is only fitting, therefore, for poor persons, who must pay in labour instead of money for the instruction they receive, or are preparing to receive. Few men who have two good shillings and four bad ones will consider them equal to four good shillings and one bad one: this, however, some of my friends do not comprehend, and prefer the chance of passing the six shillings, bad and good, to the certainty of having four good ones and only one doubtful one. It is a proposition so self-evident in my opinion, that it must prevail ultimately; and if it is not now a regulation of the College, it is not my fault. I shall not give up the effort to make it one, and will persist until I succeed in effecting it. It is frequently amusing to me to see myself abused as the supporter, nay frequently as the author, of every thing which is presumed to be exclusive, oppressive, or unjust, that either has been done or may be done by the council of the College of Surgeons. Whether this has occurred through ignorance or malevolence, I know not, and it does not signify much which; but you will also smile when you hear that, whilst I am thus designated a medical tory of the highest order by the pamphleteers, I am in doors declared to be a radical of the first order. The old gentlemen, now no more, did not hesitate to say that I had less veneration for the regulations of antiquity than Joseph Hume of radical reputation, and that I was never satisfied but in altering, and, I hope I may add, in endeavouring to amend every thing that required it; in fact, they used to call me their "Joseph Hume." When, therefore, gentlemen, you again see me abused for my exclusive principles, you also will think it not a little ludicrous, and it will be proof to you how often abuse is misapplied. I have given myself no trouble about it, satisfied the day would come, and it is not perhaps far distant, when the professional public may learn who really have been their friends.

After the first year's occupation in pharmacy, the study of medicine becomes of the first importance: it may be combined with that of surgery, but it must be followed with attention. There was an introductory lecture of mine, published some years ago with fidelity, in which you will find this opinion strongly enforced: that lecture preceded all other surgical lectures of modern date on this subject; and it is to me, therefore, that the inculcation, in these times, of the necessity of the study of practical medicine is due, in order to make a good practical surgeon. But I have done more than inculcate: I availed myself of my public situation to enforce it, and such of you as are idle, and do not like study, may blame me for causing you to spend a small portion of your time in the physician's wards of your hospital.

I am aware, gentlemen, it is said that the enforcement of attendance at an hospital is unnecessary; that it is done for the purpose of putting money into the pockets of the surgeons. Now, there may be a new road by which practical knowledge is to be acquired without seeing sick people: it may be perhaps gained by intuition, or by steam, but it has never fallen to my lot to see it obtained in any other way than by attendance at the bedside of the sick. This is not permitted in private life: I find it very difficult to take more than one student to a private patient, even in the lower ranks of life, unless they are paupers, and not often will they admit of being seen by several. It is only, then, in an hospital that a student can learn his profession, and not by running about from place to place, picking up a little here and a little there, but by a close and regular attendance at an hospital, containing a sufficient number of patients to give as frequent opportunities as is possible, or may be necessary, for obtaining good instruction. In the present state of the metropolis, an hospital to do this should contain at least 200 persons; 250 is a better number; more than 300 I think too many in one institution. I will not willingly recognise an hospital in London as competent to afford good instruction in a given small space of time, if it does not contain 200 patients. I am aware you may say to me, your own hospital contains but 103 people, yet you consider it fit to afford instruction: my reply to that may be drawn from my actions. When it was proposed to rebuild the Westminster Hospital at Charing Cross, I took a prominent part at the public meetings, but none at the private ones which preceded them: I argued on public grounds, that the city of Westminster should have at least one good hospital capable of holding 300 people; that, instead of building two small hospitals, at Charing Cross and near Westminster Abbey, it would be better to unite them, medical officers and all, and to have one hospital worthy of the city of Westminster, and capable of becoming a great school of instruction. Private vanity and private interests prevailed against public interests and public utility, and we shall have two smaller hospitals; one, the Westminster, will hold about 240 people, provided funds can be obtained to support them, which I doubt: of the other I am not competent at present to give an opinion, but the two, whatever they may be, will never be equal to what one might have been. Four years ago, I told the late treasurer of the hospital, Mr. Elliot, in order to stimulate him to exertion in rebuilding the hospital, that I should endeavour to get it struck off the list of hospitals in London considered competent for the purpose of instruction: it put him in no small passion, I assure you; but it did not prevent me, nevertheless, from moving in my place that it should be done. That it now remains a recognised hospital, is owing, I believe, to the circumstance that the rules of the College are never retrospective: it is one thing to grant a privilege, it is another to withdraw it. The new hospital will, however, soon be built, and in the mean time, as the Ophthalmic Hospital is now nearly ready for use in Chandos street, Charing cross, I shall endeavour to shew you in it a model of what I consider an hospital should be, as relates to order, cleanliness, economy, and professional education: it will be opened at Christmas.

The only proof that I am acquainted with of attendance on the sick at an hospital, is a certificate from the physicians or surgeons, as the case may be. This, I know, is made the subject of great derision, and it is said a man's knowledge is the only proof that ought to be required: I am not of that opinion, I wish to have both. I am aware of the old adage, that you may take a horse to the water, but that you cannot make him drink: nevertheless, I have seen a great many horses taken to the water, and am satisfied it is a very odd sort of a horse that will not drink, if he is taken in proper time. So it is with young men: you may make them attend an hospital, and they may not profit by it; but it is a very odd sort of a young man that does not learn something, aye, and a good deal too, if he is made to attend regularly and in proper time. If the physicians and surgeons of hospitals do not take care that their students attend regularly and at proper times, they are dishonest men; they are unworthy of their situations, and should be deprived of them. If I were satisfied that a physician or surgeon of an hospital had granted a certificate of attendance, where none, or next to none, had been given, I would endeavour to prevent the reception of any more from that individual. It is right you should be aware that I never say one thing and do another: I never grant a certificate of attendance, unless I believe it is strictly merited; I refused two this last summer, and the student must make up his time this winter, or do without it; and if, as in one instance, he succeeds in imposing on either of my colleagues, I have the power of preventing its being made use of where most necessary to them. It is the greatest advantage derived from hospital surgeons being on the court of examiners. I have now in my pocket the names of six of the students of the Westminster Hospital, who have not attended during the last four months: I shall call their names over in the theatre of the hospital, and I assure you they shall make their time good. They may attend when they please, although I would rather they came for two anatomical seasons, or two winters, than for one whole year, because I know little is done in summer, but that it is at their choice. I only wish you to understand that, having assisted in making the regulations under which you are forced to pay your money, I shall do my best to prevent your friends being cheated of it. I can make you come to the water; I acknowledge I cannot make you drink, but I believe and hope you will, and in another place I will take care you shall shew that you have: if you, therefore, wish to evade your studies, do not come to the Westminster Hospital. A certificate of attendance at an hospital for two or three winter seasons, or years, and which I have reason to believe to be a correct certificate, is very valuable to a young man, in my opinion: for many people can talk about and describe diseases they have never seen; but, when you are almost induced to believe they are very clever fellows, put them to the test, take them to the bedside of a patient labouring under the complaint in question, and their want of practical information is at once detected. Of all the students who come to London, the Aberdeen one is the best qualified to give an anatomical description of any part of the body: he will begin at the crown of the head and go down to the foot, if you please, without stop, or stay, or fault, provided you do not interrupt him; but if you do, and take him on relative situation, and that alone, he will probably be puzzled: you will detect at once how much he owes to grinding, how much to dissecting. During the war, I found no men could talk of physic half so long and half so pert as the Edinburgh M.D.; there was no disease they could not give the best account of; they had received the best possible oral instruction; but when we came to the disease in question, when we came to hard work, the poor unadorned Dublin student, who had laboured two years in the hospital and dissecting room, was worth two of them. But of all students, gentlemen, your London student, being the son of a medical man, is the worst: he is only a shade better if he happens to be the son of a physician or surgeon in the country. I do not know why this is, but I believe it is that they will not work as their fathers did before them. Let us hope, if there be any such among you, that you will prove exceptions to my observation.

